# A Method for Assessing Week-Long Cortisol Output Using a Continuously Worn Sweat Patch

**DOI:** 10.3390/mps9010013

**Published:** 2026-01-16

**Authors:** Jerrold S. Meyer, Jenna P. Blain, Karen A. Kalmakis

**Affiliations:** 1 Department of Psychological and Brain Sciences, University of Massachusetts-Amherst, Amherst, MA 01002, USA; 2 Elaine Marieb College of Nursing, University of Massachusetts-Amherst, Amherst, MA 01002, USA; kalmakis@nursing.umass.edu

**Keywords:** cortisol, HPA axis, sweat, stress, skin patch

## Abstract

Although sample matrices are available for assessing cortisol output over hours/days (serum, saliva, or urine) or months (hair or nails), there is no current method for measuring integrated cortisol output over a period of 1 week. Therefore, the primary aim of this study was to develop and validate a method for collecting and measuring sweat-derived cortisol from commercially available skin patches worn for 1 week. Additional aims were to determine whether the accumulated sweat cortisol correlated with salivary cortisol measured during the same week, and whether sweat cortisol was related to psychological stress measured using two different questionnaires. After conducting preliminary in vitro validation studies, we obtained the following data from a convenience sample of university students and employees: (a) cortisol and sodium contents of patches worn for 1 week (sodium was used to correct for variation in sweating rate), (b) mean area-under-the-curve of salivary cortisol concentrations measured for 3 days during the week of patch wearing, and (c) two different measures of psychological stress. The results demonstrate that a continuously worn sweat patch can be used to collect and measure sweat cortisol over a 1-week period. However, the patch’s cortisol contents did not correlate with either the salivary cortisol area under the curve or the participants’ psychological stress. Because previous findings showed that sweat cortisol is significantly related to both circulating and salivary cortisol levels, we hypothesize that the lack of an observed correlation between patch and salivary cortisol may have resulted from limitations of our experimental design.

## 1. Introduction

Changes in the levels and/or output of the glucocorticoid cortisol (CORT) are often used to assess hypothalamic–pituitary–adrenocortical (HPA) axis responses to acute or chronic stress. Early studies in this area typically measured CORT concentrations in blood, saliva, or urine. Somewhat more recently, investigators have exploited the knowledge that blood-borne CORT gradually accumulates over time in keratinous tissues by measuring levels of the hormone in scalp or body hair [1,2], nails [3], claws [4], and whale baleen [5] as indices of long-term CORT secretion and HPA activity.

Different sample matrices vary substantially in the time frame of HPA activity represented by their CORT content. CORT in blood or saliva provides a “point sample” of adrenocortical secretion at about the time of sample collection, providing insight into the effects of an acute stressor on the HPA axis [6]. Urinary CORT (or CORT metabolites) provides an index of adrenocortical secretion over multiple hours, typically up to a 24 h period [7]. In contrast, CORT in keratinous tissues has accumulated over many weeks, even months, which underlies the use of these sample types for assessing longer periods of adrenocortical output [8].

One potential sample matrix not mentioned in the preceding discussion is sweat excreted by eccrine glands, the most common and widely distributed type of sweat gland in the human body. To our knowledge, the first published attempt to detect CORT in sweat was a short communication by Lewis and Thorn [9]. The results were inconclusive because of the relatively nonspecific and insensitive assay method used in the study. Subsequently, Jenkins and colleagues [10] demonstrated that following an intravenous infusion of radiolabeled CORT in human participants, labeled CORT along with cortisone were detected in thermally stimulated sweat. Because sweat was collected from the entire body except for the neck and head, the samples contained secretions from both eccrine and apocrine glands. Interestingly, sweat samples contained considerably more labeled cortisone than CORT, which was the opposite finding from plasma samples obtained at the same time. A recently published abstract by Keevil and coworkers [11] confirmed a high ratio of cortisone to CORT in human eccrine sweat, similar to the ratios observed in saliva. These findings suggest the possibility of local conversion of CORT to cortisone by 11β-hydroxysteroid dehydrogenase type 2 (11β-HSD 2), a hypothesis supported by the presence of immunoreactive 11β-HSD 2 in sweat gland excretory ducts [12,13].

Concentrations of endogenous CORT in eccrine sweat have been determined by modern analytical methods such as enzyme-linked immunosorbent assay (ELISA) or liquid chromatography–tandem mass spectrometry (LC-MS/MS) [14,15,16,17]. Samples in those studies were obtained using absorbent cotton swabs or gauze pads, or by direct sweat collection into microcentrifuge tubes. However, for various reasons that likely included different collection and assay methods across studies, the mean reported values of sweat CORT were highly variable, thus complicating interpretation of the results. Moreover, to obtain sufficient sample volumes, investigators in all of those studies provoked increased sweating by subjecting participants to either aerobic exercise or a hot environment. Because both of those stressors are known to acutely activate the HPA axis [18,19], CORT concentrations in sweat under resting conditions may be lower than the values observed in these studies.

Currently, the presence of CORT in sweat is being exploited primarily by the development of wearable, hand-held, or bench-top devices designed to provide real-time indicators of HPA activity [20,21,22,23]. The major focus has been on wearable devices and their potential application to personalized medicine, particularly with respect to stress monitoring. Indeed, a few commercially developed wearable CORT sensors are now available for individual use https://www.biosens8.com/cortisol-monitor, accessed 2 January 2026; https://www.enlisense.com/corti, accessed 3 January 2026. Despite these technologies seeming to offer personalized physiological stress monitoring, published scientific studies reveal several current limitations of wearable CORT sensors. First, recent studies depicting CORT levels assessed by wearable sensors involve no more than 48 h of continuous measurement (see [21,22,23], for example). Therefore, validation has yet to be provided that wearable CORT sensors yield valid CORT levels over longer periods of time such as a week. Second, in some studies examining the ability of the sensor to track the circadian rhythm of CORT secretion, the amplitude of the depicted rhythm was much smaller than that obtained from standard salivary CORT measurements (compare sensor results from [21,22] with salivary results from [24]). Moreover, the sensors in [21,22] failed to capture the well-established CORT awakening response [24]. Third, the cross-reactivity of CORT sensors (the percent reactivity to other sweat constituents such as steroid hormones) has not been adequately tested to ensure that only authentic CORT is being measured. Of particular concern is the lack of testing for cross-reactivity to the cortisone, which, as mentioned above, is present in sweat at higher levels than CORT itself. Fourth, some sensors are not sufficiently sensitive to the small amounts of fluid excreted during passive sweating and, therefore, require iontophoresis-driven cholinergic stimulation of sweat production (e.g., [21,23]). This requirement creates two significant problems: first, the potential effect of cholinergic-mediated sweating on sweat CORT levels is unknown, and second, repeated cholinergic stimulation of the myoepithelial cells that provoke sweat secretion could produce adaptation, thereby limiting the ability of the system to provide an accurate continuous readout of CORT levels over long time periods. Finally, even for those sensors that are designed to provide a continuous dynamic readout of sweat CORT levels (e.g., [22]), data must be collected over a long time frame and subsequently integrated to ascertain chronic CORT output.

As an alternative to continuous CORT monitoring using a wearable sensor, the present study sought to leverage sweat CORT measurement to provide an index of integrated HPA activity for 1 week, a specific time period not presently covered by any method in routine use. Measuring weekly CORT output has potential application in many situations that involve ongoing changes in stress, whether increased stress such as occurs during major lifestyle changes or decreased stress that may be associated with a health-promoting intervention. Determining CORT accumulation with a sweat patch could additionally be helpful for validating a wearable CORT sensor under conditions where the sensor is being used to monitor overall CORT output week-to-week.

The use of a skin patch to assess sweat CORT accumulated over a 1-week period was previously demonstrated by Prunty and colleagues [25], who found that weekly CORT levels were consistently higher in Cushing’s disease patients than in healthy controls. These results support the hypothesis that chronic changes in circulating CORT can be demonstrated not only using hair or nail samples, but also using the sweat patch methodology. Unfortunately, the study of Prunty et al. was published only in abstract form, and few details were provided about either the researchers’ methodology or the numerical CORT data.

The aim of the present study was to develop and validate a method for collecting and measuring sweat-derived cortisol from commercially available skin patches worn for 1 week. Additional study aims were to determine whether the accumulated sweat cortisol correlated with salivary cortisol area-under-the-curve (AUC) measured during the same week, and whether sweat cortisol was related to psychological stress measured using two different questionnaires. Patches were extracted and analyzed for their contents of both CORT and sodium ion (Na^+^), which enabled us to calculate a CORT/Na^+^ ratio for each participant. This procedure was aimed at controlling for individual differences in sweat production, analogous to the use of the CORT/creatinine ratio to control for rates of urine production in urinary CORT studies [26]. The selection of Na^+^ for this purpose was based on two primary considerations: (1) small differences in sweating rate under resting conditions produce only minor changes in the sweat Na^+^ concentration [27], and (2) intra-individual sweat Na^+^ content is relatively stable as shown by an average intra-individual coefficient of variation (CV) of 16% for forearm sweat Na^+^ concentrations over three separate sampling days [28].

In addition to validating the sweat measurements, we determined whether there were any significant relationships between sweat patch CORT/Na^+^ and (1) salivary CORT diurnal area under the curve (AUC) assessed on three separate days during the same week, and (2) two different measurements of psychological stress: the widely used Perceived Stress Scale (PSS) [29] and a recently developed questionnaire called the College Student Acute Stress Scale (CSASS) [30]. The CSASS was administered only to the participants who were college students, which comprised the majority of the participant pool. We hypothesized that sweat-derived CORT would be positively correlated with each participant’s average salivary AUC value (as an index of diurnal CORT output). This hypothesis was based on the recent finding that sweat-derived CORT is highly correlated with both serum and salivary CORT, and that sweat CORT additionally tracks the daily decline in salivary CORT from morning to afternoon [31,32]. Although the literature concerning the relationship between psychological stress and various indicators of HPA activity is inconsistent [33], we additionally tested for possible correlations of sweat CORT with each psychological stress questionnaire. Lastly, participants maintained a diary in which they recorded the amount of daily water exposure (i.e., from bathing, showering, and/or swimming) during the week when their patch was worn.

## 2. Materials and Methods

### 2.1. Participants

The initial group of participants comprised a convenience sample of 50 members of the University of Massachusetts—Amherst community. The sample consisted primarily of students, but it also included a small number of staff and faculty members. Inclusion criteria for participation were: (a) at least 18 years of age, (b) agreement to abstain from all recreational substance use (including alcohol, tobacco/nicotine, cannabis, and all illicit substances) during the week of data collection, and (c) access to a home freezer for storage of saliva samples prior to delivery to the lab. Exclusion criteria were: (a) use of any corticosteroid-containing medication, (b) being pregnant, or (c) having a skin condition (e.g., eczema, psoriasis, or contact dermatitis) affecting the upper arm.

**Figure 1:**
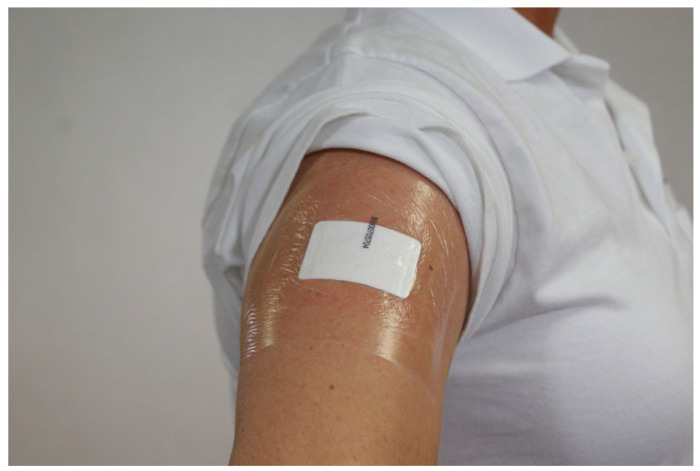
Image of a PharmChek^®^ sweat patch applied to the upper arm of a volunteer subject. Photo courtesy of PharmChek^®^.

For each participant, a patch was placed on the upper part of the non-dominant arm on day 1 according to the manufacturer’s instructions (see Figure 1). Eccrine sweat glands predominate in this area of the body, which is consistent with the use of eccrine sweat in most sweat biomarker research [34]. Participants subsequently engaged in their normal weekly activities, removed the patch on the 7th day, and then placed the patch in a plastic storage bag for later transport to the lab along with their saliva samples. Patches and saliva samples were stored at −20 °C until processing. According to the manufacturer, the PharmChek^®^ Sweat Patch (SCRAM Systems, Littleton, CO, USA) reliably adheres to the skin for 7–10 days and remains unaffected by routine showering and exercise. Nevertheless, participants were asked to complete a daily patch water exposure diary (reflecting mainly showering or bathing) for each of the days that the patch was worn. The diary consisted of five exposure categories: (1) no water exposure, (2) less than ½ h on that day, (3) ½–2 h, (4) 2–5 h, and (5) >5 h. Each participant’s water exposure score was calculated by assigning scores from 0 to 4 for the five water exposure categories, multiplying the number of days on which each score was noted, and then summing the results across the week. Sweat patch and saliva samples were collected from participants from February 2022 through April 2023, during which time each participant was tested once.

### 2.2. Sweat Patch

Sweat patches were purchased from PharmChek^®^. The PharmChek^®^ Sweat Patch consists of (a) an absorbent pad that collects sweat and retains the analytes of interest, (b) a semi-permeable polyurethane film covering the pad that permits sweat-derived water and gases to pass through into the air, and (c) an adhesive backing to maintain contact between the patch and the skin (https://www.pharmchek.com/resources/how-the-sweat-patch-works; accessed 9 January 2025). Although the sweat patch was designed to be used for forensic drug testing, validation studies described below showed that sweat-derived CORT is efficiently captured by the patch and can be eluted for quantitative analysis. We reasoned that patch CORT content would be influenced not only by adrenal output, but also by the amount of sweat captured by the patch. Therefore, we eluted and measured patch Na^+^ content as a proxy for sweat accumulation and corrected each CORT value by dividing that value by the corresponding amount of recovered Na^+^. As mentioned earlier, the correction of each patch’s CORT content by its corresponding Na^+^ content is analogous to the standard practice of correcting urinary CORT levels using urinary creatinine (CORT/creatinine ratio) [26].

### 2.3. Salivary Cortisol

We sought to determine whether weekly CORT output measured using the sweat patch was significantly related to the AUC of diurnal salivary CORT, a widely used measure of daily CORT output [35]. To accomplish this, each participant was given 15 sets of collection materials from Salimetrics (Carlsbad, CA, USA) to obtain saliva samples using the passive drool method. The calendar of daily study activities is shown in the Supplementary Table S1. Briefly, participants were instructed to collect five samples each on days 2, 4, and 6 of the week during which they were wearing the patch. Targeted sampling times were (1) waking (7:00–8:00 AM), (2) 30 min after waking, (3) midday before lunch (12:00–1:00 PM), (4) late afternoon (between 4:00 and 5:00 PM), and evening (9:00–10:00 PM). Actual collection times were recorded for each sample. Samples were frozen by the participant in home appliances and then later transported to the laboratory along with the corresponding sweat patch for storage at −20 °C until assay. For analysis, saliva samples were thawed, centrifuged, and then analyzed using the Salimetrics CORT ELISA kit according to the manufacturer’s directions. High and low CORT controls provided in the kit were included in each assay. Across all runs, the intra-assay CV was 3.63% and the inter-assay CVs were 5.29% and 12.06% for the high and low controls, respectively. According to the manufacturer, percent cross-reactivity of the anti-CORT antibody to other steroid hormones is as follows: dexamethasone (synthetic steroid) = 19.2%; prednisolone = 0.57%; corticosterone = 0.21%; 11-deoxycortisol = 0.16%; cortisone = 0.13%; all others < 0.1% or not detected. CORT AUC on each sampling day was determined by the trapezoidal method using Prism v. 8.30 software (GraphPad, Boston, MA, USA).

### 2.4. Psychological Stress

On day 7 of the study, participants completed two online instruments to assess their psychological stress. The first instrument was the Perceived Stress Scale (PSS), a well-established 10-item questionnaire that assesses feelings of stress over the previous month [29]. Each item is scored on a 5-point Likert scale ranging from 0 (Never) to 4 (Very Often), and each participant’s PSS score is summed across the 10 items. According to the standard interpretation of PSS scores, 0–13 represents low stress, 14–26 represents moderate stress, and 27–40 represents high stress. The second instrument was the College Student Acute Stress Scale (CSASS), a questionnaire recently developed by researchers at the University of Massachusetts to specifically assess 14 types of stress commonly experienced by college students [30]. Each item is scored on a 5-point Likert scale ranging from 0 (No Stress) to 4 (Constant Stress), and each participant’s CSASS score is summed across the 14 items. The time frame encompassed by the CSASS is the week prior to questionnaire completion, thus aligning with the week in which the sweat patch was worn and salivary CORT was collected. Because the CSASS targets stressors specific to college students, it was completed only by the student participants in the study.

### 2.5. Sweat Patch Extraction

Several individual solvents and solvent combinations were tested for their ability to elute both CORT and Na^+^ from skin patches. We ultimately determined that the best choice was a 1:1 mixture of reagent-grade ethanol/HPLC-grade water based on its ability to recover quantitatively both CORT and Na^+^ from patches (see validation experiments below).

For extraction, each patch was cut approximately into quarters using a clean surgical scissors and the resulting pieces were carefully placed at the bottom of a standard 20 mL glass liquid scintillation vial. Three ml of the ethanol/water extraction solvent was added to the vial and incubated for 30 min at room temperature on an orbital shaker at 100 Hz. Following the incubation, 1.2 mL of the extract was removed and transferred to a 20 mL glass screw-cap tube with a PTFE-lined cap (preliminary studies showed that because of liquid absorption by the patch, it was difficult to routinely recover more than this volume of extract). Ten volumes (12.0 mL) of HPLC-grade dichloromethane were added to each glass tube, after which the tube was vortexed vigorously for 10 s and then shaken on a nutator for 10 min. Tubes were centrifuged at 1000 RPM for 5 min to separate the aqueous and organic layers. Two hundred µL of the upper aqueous layer was removed using a disposable glass Pasteur pipet and transferred to a microcentrifuge tube for measuring Na^+^ content, after which the remainder of the aqueous layer was aspirated and discarded. The organic phase containing CORT was evaporated at 50 °C under a stream of air. The resulting dried extract was reconstituted in 165 µL of Salimetrics assay diluent with vigorous vortexing. After reconstitution, the extract was transferred to a Costar Spin-X filter tube (0.45 µm cellulose acetate filter), filtered to remove any residual solid material, and then stored frozen at −20 °C for later CORT assay.

CORT analyses were performed using the same Salimetrics kit as for the saliva samples, and each participant’s patch extract was analyzed in the same run as their saliva samples. Patch Na^+^ content was determined with a Horiba LAQUAtwin-Na-11 sodium ion meter (Horiba Instruments, Irvine, CA, USA) using the recommended sampling sheet B Y046 for small sample measurement. Fifty µL of the extracted aqueous layer was applied to the sampling sheet to obtain the Na^+^ concentration, after which the sheet was removed, and the meter was rinsed with HPLC-grade deionized water and dried in preparation for the next sample. Before each set of measurements, the meter was calibrated according to the manufacturer’s instructions. Finally, meter Na^+^ readouts in parts per million (PPM) were converted to µmol Na^+^ before statistical calculations were performed.

### 2.6. Validation Experiments

Before conducting the main study, in vitro validation experiments were performed to determine the recovery of both CORT and Na^+^ using the ethanol/water elution solvent. For CORT recovery, 0.01 µCi of [^3^H]cortisol in ethanol (New England Nuclear, Boston, MA, USA) was applied to clean patches and allowed to dry. Patches were then extracted using our standard procedure and the amount of [^3^H]cortisol in the extract was measured. Next, we performed a serial dilution test for which extracts from several patches worn by participants were combined to yield a concentrated patch extract. The dilution test was conducted by analyzing the undiluted extract along with five different dilutions ranging from 80% of the original extract to 10%.

For validation of Na^+^ measurements, we first prepared a solution of artificial sweat containing 327 mM ammonium chloride, 166 mM lactic acid, 83 mM urea, 42 mM acetic acid, and 34 mM sodium chloride, adjusted to pH 4.7 with 2 N sodium hydroxide [36]. To assess Na^+^ elution using our standard extraction procedure, clean patches were treated with deionized water, or four or five different volumes of artificial sweat ranging from 0.15 mL to 0.75 mL. Patches were subsequently dried, extracted as usual, and their Na^+^ content measured.

### 2.7. Statistical Analyses

The three biomarkers measured in the study (patch CORT, patch Na^+^, and mean salivary CORT AUC) were first tested for outliers using the Grubbs’ test. The test identified an extreme outlier in the CORT content from one participants’ patch, which resulted in the exclusion of that individual’s data. Next, the distribution of each biomarker’s values was tested for normality using multiple tests (Anderson-Darling, D’Agostino and Pearson, Shapiro–Wilk, and Kolmogorov–Smirnov), and in every case, all four tests determined that the three biomarkers were not normally distributed. As a result, we performed a log_10_ transformation on those values, and the log-transformed data were used for all subsequent statistical tests. To normalize the patch CORT content by patch Na^+^ content, we subtracted the log Na^+^ value in µmol from the log CORT value in pg (subtraction is used to calculate a ratio between two log values). Finally, Pearson-Product moment correlations were performed to determine whether there were any significant relationships between (1) log CORT/Na^+^ ratio and log mean salivary CORT AUC, (2) log CORT/Na^+^ ratio and PSS score, and (3) log CORT/Na^+^ ratio and CSASS score. All statistical procedures were performed using GraphPad Prism.

## 3. Results

### 3.1. Validation Experiment Results

**Figure 2:**
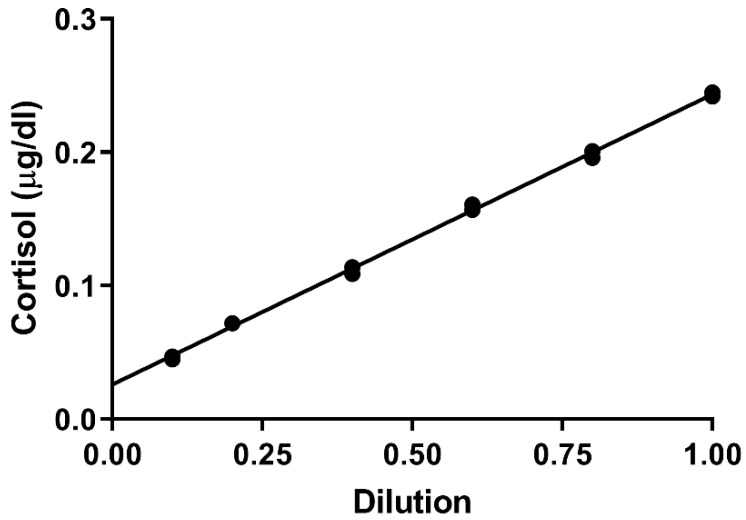
Linear regression from a single representative sweat patch CORT dilution test in which measured CORT concentrations were related to the dilution of the original concentrated patch extract represented by 1.00 (R^2^ = 0.998) Note that the data points plotted on the graph are values from duplicate wells on the same plate.

When radiolabeled CORT was applied to a clean patch and extracted using our standard procedure, the measured recovery was 95.9% ± 1.7% (mean ± SEM; N = 4). These results showed virtually complete recovery of CORT using the ethanol/water mixture followed by dichloromethane back-extraction. The second validation test involved serial dilution of a concentrated sweat patch extract, CORT measurement of each dilution, and then a linear regression analysis of the CORT values. This analysis yielded a highly significant linear trend with an R^2^ of 0.977 ± 0.009 (mean ± SEM; N = 3). Note that the intersection of the regression lines with the y-axis was consistently offset from 0 by a small amount, as shown by the example results in Figure 2. Thus, it appears that the Salimetrics ELISA kit slightly overestimated the CORT content of each sweat patch. However, this effect should have relatively little impact on inter-individual or inter-group comparisons of sweat patch CORT measurements.

**Figure 3:**
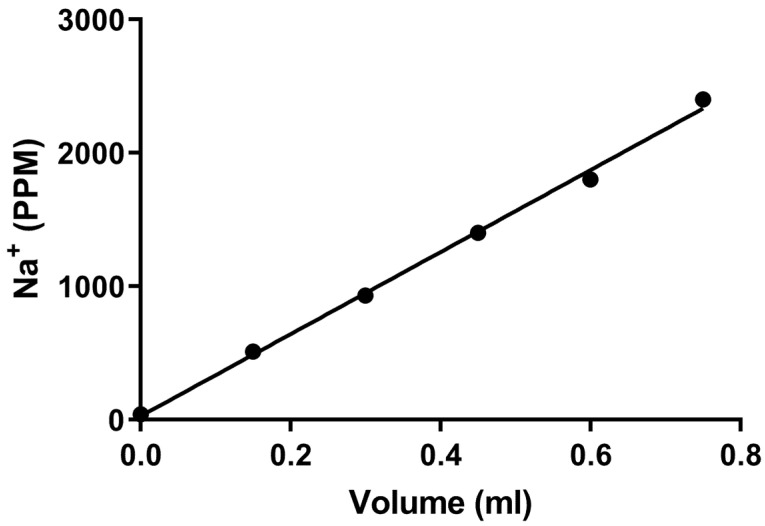
Linear regression from a single representative sweat patch Na^+^ recovery test in which the amount of measured Na^+^ was related to the volume of artificial sweat deposited on the patch (R^2^ = 0.997).

In the Na^+^ recovery test using different volumes of artificial sweat, recovered Na^+^ values in PPM were highly linear, with an R^2^ of 0.984 ± 0.004 (mean ± SEM; N = 5; see Figure 3 for a representative result). This result confirms that the Na^+^ measurement obtained from each patch was a reliable indicator of the amount of Na^+^ deposited in the patch from the participant’s sweat.

### 3.2. Sweat Patch and Salivary CORT Results

From the original 50 participants, 11 were eliminated from the final data analyses for several reasons. Three participants did not provide usable saliva samples, five had patches that did not yield sufficient amounts of CORT and/or Na^+^ for reliable measurement (this mostly occurred early in the study, after which we reduced the reconstitution volume of the patch extract prior to CORT and Na^+^ analyses), one turned in a wet patch, one had a patch CORT content that was found to be an extreme outlier, and one chose not to fill out the psychological stress questionnaires. The individual with the extreme patch CORT content had representative salivary CORT levels, raising the possibility of external contamination of the patch by that participant. The included 39 participants (32 female) had an age range of 18 to 63 (25.1 ± 1.9, mean ± SEM).

Table 1 presents descriptive statistics for the sweat patch CORT and Na^+^ measurements and the salivary CORT AUC calculations. Note the wide range of values for each of these variables, a finding that is further discussed below.

To determine whether patch CORT contents were influenced by participant water exposure, we performed a correlational analysis between log CORT/Na^+^ ratio and self-reported water exposure during the week when the patch was worn. The results indicated a negligible relationship between the variables (r = 0.03, df = 37, NS), which is consistent with the manufacturer’s contention that the sweat patches are unaffected by routine water exposure.

**Figure 4:**
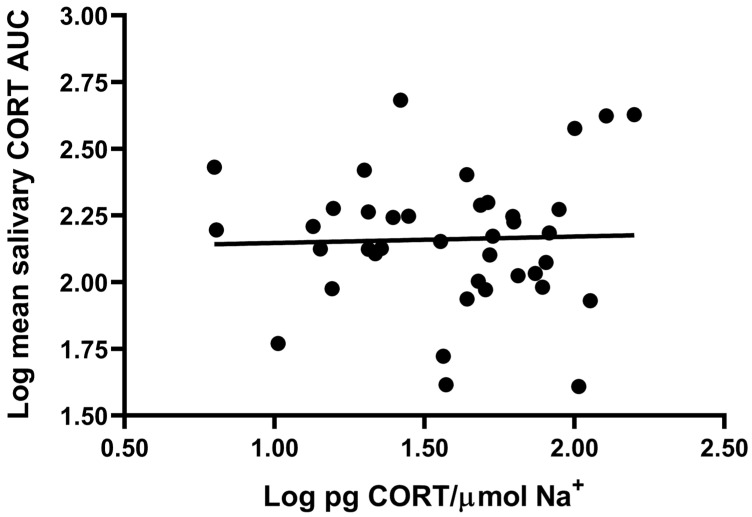
Correlation between log sweat patch CORT/Na^+^ and log mean salivary CORT AUC across participants (r = 0.03, df = 37, NS).

We tested the hypothesis that CORT accumulated in the sweat patch over 7 days was related to average CORT output over the same time period by performing a correlational analysis between log CORT/Na^+^ and log salivary CORT diurnal AUC averaged over 3 days as an index of CORT output. The hypothesis was not supported, as there was no significant relationship between normalized patch CORT content and mean salivary CORT diurnal AUC (r = 0.03, df = 37, NS; Figure 4).

### 3.3. Psychological Stress and Sweat Patch CORT Results

**Figure 5:**
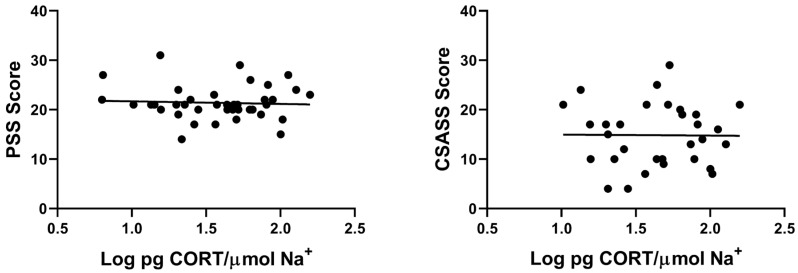
Correlation between log sweat patch CORT/Na^+^ and PSS score (**left panel**; r = 0.05, df = 37, NS) or CSASS score (**right panel**; r = 0.01, df = 29, NS) across participants. Only the student participants completed the CSASS questionnaire.

For the psychological stress measures, all 39 participants completed the PSS with a mean ± SEM of 21.4 ± 0.6 and a range of 14–31 (possible scores range from 0 to 40). The 31 participants who were college students provided CSASS responses with a mean ± SEM of 14.8 ± 1.1 and a range of 4–29 (possible scores range from 0 to 56). A correlation analysis was performed to ascertain whether patch CORT accumulation determined by log CORT/Na^+^ was related to participants’ psychological stress measured using either the PSS or CSASS. Figure 5 shows that neither PSS nor CSASS scores were correlated with log CORT/Na^+^ values (r = 0.05, df = 37, NS for PSS; r = 0.01, df = 29, NS for CSASS).

## 4. Discussion

The present study was designed to develop and validate a method for collecting and measuring sweat-derived CORT from commercially available skin patches worn for a period of one week. Additional study aims were to determine whether the accumulated sweat CORT correlated with salivary CORT AUC measured during the same week, and whether sweat CORT was related to psychological stress measured using two different questionnaires. The results demonstrated that sweat-derived CORT could be collected and analyzed in most participants, especially after the sample reconstitution volume was reduced in order to increase the CORT concentration prior to assay. An extraction solvent of 1:1 ethanol/water fully eluted both CORT and Na^+^ from the patch, and the CORT/Na^+^ ratio could potentially be used to account for individual differences in sweat production. However, the CORT/Na^+^ ratio was not correlated with either salivary CORT AUC or psychological stress.

In recent years, sweat analysis has become a major area of interest because of its potential applications to diverse areas including clinical diagnosis and stress monitoring [34,37]. Although most investigators have focused on sweat monitoring using wearable sensors, the information presented in the Introduction demonstrates that these devices still have significant limitations. One such limitation is that, similar to salivary or plasma CORT measurements, many readings must be taken over time by the sensor and subsequently integrated in order to assess chronic CORT output. We reasoned that an integral of weekly CORT output obtained by means of sweat patch collection could be valuable for monitoring week-to-week changes in *chronic* HPA activity in situations such as stressful lifestyle changes, progression and treatment of a major somatic or psychiatric illness, or the progress of recovery during and after a stress-reduction intervention. This reasoning is analogous to the now-routine use of hair CORT to determine long-term changes in average CORT output in preference to the measurement of CORT in short-term biological samples such as plasma, saliva, or urine.

If weekly sweat CORT measurements accurately reflect adrenal output over that same time period, then the measurements should be correlated with a standard measure of adrenocortical activity such as diurnal salivary CORT AUC determined during the week of patch use. A similar approach has been used to help validate hair CORT levels as an index of monthly CORT output [38]. Surprisingly, no such relationship was observed in the present study. It is possible that sweat CORT content is not indicative of circulating levels of the hormone. However, this seems unlikely because of the previously mentioned findings that sweat CORT measured using a real-time electrochemical sensor patch correlated highly with both serum and salivary CORT [31] and that individuals with Cushing’s disease compared to healthy controls accumulated more CORT in a skin patch worn for a week [25]. Moreover, Grass and coworkers [15] found high correlations between sweat CORT and salivary CORT AUC in participants subjected to either treadmill- or sauna-induced sweat production. The lack of a relationship between sweat and salivary CORT in the present study could be due, in part, to the large amount of both intra- and inter-individual variability in both measures. For example, Table 1 shows that the untransformed CORT/Na^+^ ratios among participants varied across a 25-fold range. Another way of expressing this variability is the CV of these ratios, which was over 71%. Salivary CORT measures also showed high CV values, specifically 61.1% for mean CORT AUC and 52.4% for mean waking salivary CORT concentrations. High variability in sweat patch CORT could be related to a variety of factors, including individual differences in CORT diffusion from the bloodstream into the sweat glands, variation in CORT conversion to cortisone within the glands, and variable adhesion of patches to the participants’ skin. On the other hand, variability in salivary CORT measures may be related to the inconsistent sleep–wake patterns of the participants, as discussed below in the limitations section.

We also found no relationship between sweat patch CORT and two different measures of psychological stress. While some studies have observed significant correlations between psychological stress and CORT measured in various sample matrices (e.g., saliva or hair), many have not observed this relationship [33,39]. Indeed, in the case of hair samples, Weckwesser and colleagues [40] determined that relatively little variance in hair CORT could be attributed to variance in psychological stress measures. In the present study, the CSASS questionnaire covered the same 1-week time frame as the sweat collection, whereas the PSS covered the 1-month period prior to questionnaire completion. A temporal mismatch could, therefore, have contributed to the lack of correlation between sweat CORT accumulation and PSS scores, but it cannot account for the same lack of correlation with the CSASS.

The present study had several limitations that may have contributed to the lack of a relationship between sweat patch CORT and salivary CORT AUC. The first limitation relates to the previously mentioned variability in mean AUC values within the study sample, which consisted largely of undergraduate students. This was likely due not only to normal inter-individual variation in CORT dynamics, but also to a large variation in reported wake-up times (ranging from 5:10 AM to 12:00 PM) among the participants. Presumably because of academic schedule restrictions, there was also a lot of variability in the other daytime saliva collection times. These factors may have limited the ability of our AUC calculations, despite being averaged over three separate days, to accurately reflect daily CORT output. Future attempts to validate sweat patch CORT as an index of integrated CORT output should either ensure reliable timing of saliva collection (especially in the early morning) or use an alternative approach such as measurement of urinary CORT excretion [41,42]. A second limitation involves the use of patch Na^+^ content to control for the quantity of sweat collected by a given patch. We deemed it necessary to correct patch CORT values for individual differences in sweating rate, and Na^+^ was selected as the most appropriate sweat component for this purpose. However, we acknowledge several problems with using Na^+^ concentration as a proxy for sweating rate. For example, although sweat Na^+^ concentration showed only modest variability at low (resting) sweating rates, the concentration did increase as a function of sweating rate when sweating was provoked by vigorous exercise in a warm environment [27]. Although we did not collect any exercise information from our participants, to our knowledge none of them were involved in collegiate athletics. On the other hand, modest inter-individual differences in sweating rate could have influenced sweat Na^+^ concentrations among the participants. Other studies have found that sweat Na^+^ concentration can vary as a function of hydration status, dietary Na^+^ consumption, and heat acclimation [43,44]. Together, these factors likely added a certain amount of variability to the CORT/Na^+^ ratio used in our calculations. Other sweat metabolites such as lactate or urea could be considered as alternatives to Na^+^, but the available evidence suggests that these compounds are no better choices as proxies for sweating rate [44]. Because most of the participants were females, a third limitation concerns the possibility that sweat and salivary CORT may have been influenced by phase of the menstrual cycle, thereby adding uncontrolled variability to the data. In a study using salivary CORT measurements, Montero-López and colleagues [45] found that baseline CORT and the CORT response to stress were both elevated in women during the follicular phase of the cycle compared to the luteal phase. A fourth limitation concerns the possibility that the outer layer of the cellulose absorbent pad of the sweat patch functions as a semi-permeable membrane that could permit the development of a CORT concentration gradient across the membrane. In this hypothetical scenario, the accumulation of CORT in the patch produces a concentration higher than the CORT concentration in newly excreted sweat, impeding the further diffusion of the sweat-borne CORT from the skin into the patch. Although we could find no studies in the literature testing for this possibility, its plausibility arises from the fact that such a concentration gradient operating in the reverse direction forms the basis of transdermal drug delivery using skin-adhering patches [46]. A fifth limitation was the use of an immunoassay to measure patch CORT concentrations. Although the serial dilution test we performed supported the notion that the assay was measuring authentic CORT in the patch extracts, sensitivity limits of the immunoassay likely contributed to our inability to detect measurable amounts of patch CORT for several participants. Future studies could benefit from the use of more sensitive and selective assay methods such as liquid chromatography–tandem mass spectrometry (LC-MS/MS). The use of LC-MS/MS would also facilitate the simultaneous analysis of both CORT and cortisone in the patches. Because of the previously mentioned findings of more cortisone than CORT in sweat, measuring both steroids in sweat patches could be quite valuable by providing data on each individual steroid’s concentrations, cortisone/CORT ratios, and total glucocorticoid sweat accumulation. Finally, because this was a methodological study, we made no attempt to compare stressed and unstressed individuals. Perhaps the sweat patch method used here would be more useful in detecting stress-related differences in CORT output than in measuring baseline CORT levels.

## 5. Conclusions

The present study describes the development and validation of a procedure to measure sweat CORT output over a 1-week period using an inexpensive, commercially available sweat patch. Quantitative recovery of CORT and Na^+^ from the patch was demonstrated, as was a linear function obtained from serial dilutions of a concentrated patch extract. On the other hand, patch CORT contents corrected for Na^+^ concentration (as an index of sweating rate) did not correlate with average weekly CORT output based on the mean of 3 days of salivary CORT AUC assessed during the same week that the patch was worn. Additionally, patch CORT/Na^+^ ratios were not correlated with two different measures of psychological stress. Thus, despite the potential utility of measuring weekly integrated CORT output using eccrine sweat as the biofluid of interest, more research is needed to determine whether such measurements represent a valid indicator of HPA axis activity under either resting or stressful conditions.

## Figures and Tables

**Table 1:** Descriptive Statistics of CORT and Na^+^ Measures (untransformed).

Patch Cortisol Content (pg)	Patch Na^+^ Content (µmol)	Cortisol/Na^+^ Ratio	Mean Salivary Cortisol AUC (µg/dL·min) ^a^
562.3 ± 103.2 (26–3605)	20.88 ± 5.61 (1.95–137.0)	50.38 ± 5.77 (6.30–158.33)	170.3 ± 16.7 (40.7–482.0)

Values shown are mean ± SEM (range). ^a^ Cortisol AUC means are based on three separate determinations except for four participants who only provided two sets of saliva samples.

## Data Availability

The original data from this study will be made available by the corresponding author upon reasonable request.

## Supplementary Materials

The following supporting information can be downloaded at: https://www.mdpi.com/article/10.3390/mps9010013/s1, Table S1: Participant Calendar of Daily Activities.
