# Supplementary material for: A Method for Assessing Week-Long Cortisol Output Using a Continuously Worn Sweat Patch
Source: Methods Protoc. 2026 Jan 16;9(1):13. doi: 10.3390/mps9010013 (PMC12821472; doi:10.3390/mps9010013)
Supplement: Supplementary file 1 [file mps-09-00013-s001.zip › mps-4068070-supplementary.pdf]

**Supplementary Table S1. Participant Calendar of Daily Activities**

**Day 1** – Apply the sweat patch
Complete water exposure diary

**Day 2** – Collect **5** saliva samples at the following times:
When you awake (between 7 AM and 8 AM)
30 minutes after you awake (between 7:30 AM – 8:30AM)
Midday (between 12:00PM and 1:00PM)
Late afternoon (between 4:00PM and 5:00PM)
Evening (between 9:00PM and 10PM)
Complete water exposure diary

**Day 3** – Complete water exposure diary

**Day 4** - Collect **5** saliva samples at the following times:
When you awake (between 7 AM and 8 AM)
30 minutes after you awake (between 7:30 AM – 8:30AM)
Midday (between 12:00PM and 1:00PM)
Late afternoon (between 4:00PM and 5:00PM)
Evening (between 9:00PM and 10PM)
Complete water exposure diary

**Day 5** – Complete water exposure diary

**Day 6** - Collect **5** saliva samples at the following times:
When you awake (between 7 AM and 8 AM)
30 minutes after you awake (between 7:30 AM – 8:30AM)
Midday (between 12:00PM and 1:00PM)
Late afternoon (between 4:00PM and 5:00PM)
Evening (between 9:00PM and 10PM)
Complete water exposure diary

**Day 7** – Remove the patch at approximately the same time you applied it.
Complete water exposure diary
Complete **Online Questionnaires.**
